# Data on nation-wide activity in intensive cardiac care units in France in 2014

**DOI:** 10.1016/j.dib.2017.05.018

**Published:** 2017-05-19

**Authors:** François Roubille, Grégoire Mercier, Clément Delmas, Stéphane Manzo-Silberman, Guillaume Leurent, Meyer Elbaz, Adeline Riondel, Eric Bonnefoy-Cudraz, Patrick Henry, For the French group of ICCU, Member of the Société Française de Cardiologie, Member of the ACCA association, Member of the ESC

**Affiliations:** a Cardiology Department, University Hospital of Montpellier, Montpellier, France; b PhyMedExp, University of Montpellier, INSERM U1046, CNRS UMR 9214, 34295 Montpellier cedex 5, France; c Economic Evaluation Unit at Montpellier Teaching Hospital, University of Montpellier, Montpellier, France; d Intensive Cardiac Care Unit, Cardiology Department, University Hospital of Rangueil, Toulouse, France; e Department of Cardiology, Inserm U942, Lariboisière Hospital, AP-HP, Paris Diderot University, Paris, France; f CHU Rennes, Service de Cardiologie et Maladies Vasculaires, Rennes F-35000, France; g Hospices Civils de Lyon, Université Claude Bernard Lyon 1, Lyon, France

**Keywords:** Acute cardiac care, Database, Emergency, Intensive cardiac care unit, Intensive cardiovascular care unit, Intensive care

## Abstract

We present data in relation to the article entitled “Description of acute cardiac care in 2014:

A French nation-wide database on 277,845 admissions in 270 ICCUs”.(10.1016/j.ijcard.2017.04.002) (Roubille et al., 2017) [1]: the main characteristics of the pathologies managed in the intensive cardiac care units (ICCUs), the details on the interventions performed and the main differences between centers following the size of the centers and a figure presenting the monthly variations of admissions in the ICCUs in France in a total of 277,845 patients in 270 centers admitted at least one time in the ICCUs in 2014 (exhaustive data).

**Specifications Table**

**Table:** 

Subject area	*Medicine*
More specific subject area	*Acute Cardiac care*
Type of data	*Table, Figure*
How data was acquired	*Statistical analysis*
Data format	*Word*
Experimental factors	*Not applicable*
Experimental features	*Not applicable*
Data source location	*National French database*
Data accessibility	*Available in the present manuscript as well as in the main manuscript*[1]

**Value of the data**

• Main characteristics of the pathologies managed in the intensive cardiac care units (ICCUs).
• The details on the interventions performed, allowing comparisons with other databases.
• Main differences between centers following the size of the centers, providing interesting features for health-system policies.
• Monthly variations of admissions, urging for adaptations.

## 1 Data

### 1.1 Background

The intensive cardiac care unit (ICCU) has greatly evolved for decades: it is no longer only patients with coronary artery disease (CAD). The clinical characteristics and pathological profiles of patients have markedly changed. Detailed data on the topic are critically lacking.

Data provided:

• main characteristics of the pathologies managed in the intensive cardiac care units (ICCUs),
• the details on the interventions performed
• main differences between centers following the size of the centers
• monthly variations of admissions

## 2 Experimental design, materials and methods

### 2.1 Data sources

Data are derived from an administrative database: French public and private hospitals are financed through a prospective payment scheme based on diagnosis-related groups (DRGs) [2]. The national hospital discharge database (Programme de Médicalisation des Systèmes d’Information) includes data from all public and private hospitals, and for all payers. Data includes diagnoses (encoded using the International Classification of Diseases, Tenth Revision – ICD-10), procedures (encoded using the French Classification Commune des Actes Medicaux – CCAM), age, sex, admission and discharge status, provider, French DRGs, and department level (an administrative division (*n*=96)). As all public and private hospitals receive additional service coverage, all ICCU admissions are recorded in the discharge database.

Demographic data on the French population is from the 2014 French census.

### 2.2 Variables

We included all inpatient stays of adult patients with at least one ICCU admission during the year 2014.

Diagnoses were grouped into clinically meaningful categories based on ICD-10 codes, whether as principal or secondary diagnosis (see methodology [1]).

The severity level is included in the discharge database and takes into account the patient׳s age, various cardiovascular pathologies and complications occurring during the admission.

Mortality was assessed on in-hospital death, which is included in the discharge database.

The centers were grouped into 4 categories according to the volume of patients admitted based on the quartiles of the observed distribution.

Population-based ICCU admission rates of patients above 75 were calculated at the departmental level.

Importantly, we present here exhaustive data representing 100% of the ICCUs in France and of the patients admitted in ICCUs.

### 2.3 Statistical analysis

Preliminary descriptive analysis included frequencies for categorical variables, means±SD, and medians (minimum-maximum) for continuous variables.

Categorical variables were compared by a chi squared test. Comparisons of continuous variables were performed by Mann-Whitney, ANOVA or Kruskall-Wallis tests as appropriate and completed by the Bonferroni correction.

To determine the relative importance of the predictor variables on in-hospital mortality, a multivariate analysis using logistic regression was performed. A backward selection of the variables was used. Age, gender, center size and diagnosis were entered in the model. Adjusted odds-ratio (OR) and their confidence intervals (CI) were calculated.

All statistical tests were two-sided and a *p* value of less than 0.05 was considered statistically significant

All analyses were performed using SAS software, version 9.2.

## Manuscript for the Data in Brief article

Table 1A and B. Patients admitted with CAD comprised the largest group (49.0%) of admissions. Acute CAD was the main cause (39.3%) for admission, with patients significantly younger in age (mean age 67.4 y), primarily male (69.2%), and had significantly better outcomes (mortality 4.0%) and shorter hospital stays (mean stay 6.7 d). The second largest category for admissions was patients with arrhythmias (15.2%). Patients with heart failure (HF) represented 10.0% of admissions. HF patients were significantly older (mean age 75.2 y), hospitalized significantly longer (mean stay 12.0 d) and had significantly poorer outcomes (mortality 10.5%) than the other types of admission. Valvular disease was the fourth cause of admission (Table 2, Table 3).

**Table 1:** Main characteristics of the pathologies managed in the ICCUs.

A. Admission cause only (Principal diagnoses)		
Disease	N	%
Coronary disease	136,279	49.0
Arrhythmias^a^	42,161	15.2
Heart failure	27,828	10.0
Valvular diseases	6243	2.2
Infectious diseases	597	0.2
Others	64,737	23.4

B. Pathologies presented during hospitalization						
Disease group	N	%	Stay duration (mean)	Mean age	Mortality (%)	Sex ratio (% men)
Acute CAD	109,342	39.3	6.7	67.4	4.0	69.2
Arrhythmias^a^	106,432	38.3	9.6	72.4	7.8	59.8
Heart failure	77,554	27.9	12.0	75.2	10.5	58.0
Valvular diseases	37,724	13.5	11.4	76.1	7.2	54.6
*P*-values			<0.001	<0.001	<0.001	<10^-6^

CAD: coronary artery disease.

The diseases are grouped logically (see Section 2). The total amounts can be >100%.

a Including 22,903 supraventricular arrhythmias (8.2% of total events patients); 11,924 conduction disorders (4.3%); 3546 ventricular arrhythmias (1.3%); 1427 cardiac arrests (0.5%).

**Table 2:** Interventions realized.

Intervention	N	Percentage of total number of interventions (%)
Coronary intervention	102,279	70.8
Pacemaker/automatic implantable defibrillator	20,438	14.1
Arrhythmias	7042	4.9
Valvular surgery	6221	4.3
Coronary bypasses	4.097	2.8
Others	4461	3.1
Total	144,538	100

**Table 3:** Main differences between centers following the size and the interventional capability of the centers.

A. Size						
Size of the center	Age	Sex ratio	Mortality	Stay duration	% CAD	% HF
Q1	71,18	58,40	5.8	8.5	27.9	34.5
Q2	69,82	61,50	4.7	7.6	39.1	28.2
Q3	68,81	63,89	4.2	6.8	42.5	25.7
Q4	67,51	64,13	4.8	7.8	40.6	27.3
*P*-value	<.001	<10^−6^	<10^−^^6^	<.001	<10^−6^	<10^−^^6^

B. Interventional capability						
Interventional capability	Age	Sex ratio	Mortality	Stay duration	% CAD	% HF
No	72.25	56.26	5.7	8.59	22.63	36.43
Yes	68.28	63.64	4.4	7.51	41.05	27.09
*P*-value	<.001	<.001	<.001	<.001	<.001	<.001

Q1–4: quartiles 1–4.

## Figures and Tables

**Fig. S1:**
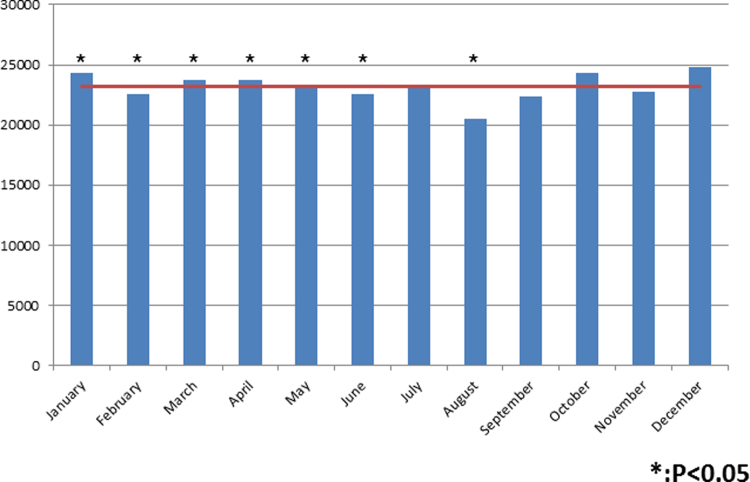
monthly variations of admissions in ICCUs. .
