# Supplementary material for: Data on nation-wide activity in intensive cardiac care units in France in 2014
Source: Data Brief. 2017 May 19;13:166–70. doi: 10.1016/j.dib.2017.05.018 (PMC5451179; doi:10.1016/j.dib.2017.05.018)
Supplement: Supplementary file 1 — Supplementary material [file mmc1.docx]

**Conflicts of interest form**

The authors declare no conflict of interest in relation with this work.
